# Highly Visible Light Responsive, Narrow Band gap TiO_2_ Nanoparticles Modified by Elemental Red Phosphorus for Photocatalysis and Photoelectrochemical Applications

**DOI:** 10.1038/srep25405

**Published:** 2016-05-05

**Authors:** Sajid Ali Ansari, Moo Hwan Cho

**Affiliations:** 1 School of Chemical Engineering, Yeungnam University, Gyeongsan-si, Gyeongbuk 712-749, South Korea

## Abstract

This paper reports that the introduction of elemental red phosphorus (RP) into TiO_2_ can shift the light absorption ability from the UV to the visible region, and confirmed that the optimal RP loading and milling time can effectively improve the visible light driven-photocatalytic activity of TiO_2_. The resulting RP-TiO_2_ nanohybrids were characterized systematically by a range of techniques and the photocatalytic ability of the RP-TiO_2_ photocatalysts was assessed further by the photodegradation of a model Rhodamine B pollutant under visible light irradiation. The results suggest that the RP-TiO_2_ has superior photodegradation ability for model contaminant decomposition compared to other well-known photocatalysts, such as TiO_2_ and other reference materials. Furthermore, as a photoelectrode, electrochemical impedance spectroscopy, differential pulse voltammetry, and linear scan voltammetry were also performed in the dark and under visible light irradiation. These photoelectrochemical performances of RP-TiO_2_ under visible light irradiation revealed more efficient photoexcited electron-hole separation and rapid charge transfer than under the dark condition, and thus improved photocatalytic activity. These findings show that the use of earth abundant and inexpensive red phosphorus instead of expensive plasmonic metals for inducing visible light responsive characteristics in TiO_2_ is an effective strategy for the efficient energy conversion of visible light.

The design of a photocatalyst for the utilization of solar energy is considered one of the most promising and clean approaches towards pollutant removal from aquatic environments. Metal oxide-mediated photocatalysis under UV light and/or visible light have been reported widely in terms of stability, efficiency and metal free photocatalysts^1^,^2^,^3^,^4^. Among them, TiO_2_ is a more popular photocatalyst for the conversion of solar energy, hydrogen generation via water splitting reaction and dye degradation because of its stability, high catalytic activity, low cost, easy availability etc.^3^,^4^. On the other hand, the catalytic efficiency of TiO_2_ in the wider spectrum of solar energy is still low because of its wide band gap and high recombination rate of photoinduced electrons and holes. Several attempts have been made to enhance the photocatalytic activity and extend the absorption edge of TiO_2_ to the visible region via metal and non-metal doping, anchoring of noble metals, textural designing and surface defect engineering^2^,^3^,^4^,^5^. On the other hand, the catalytic efficiency and absorption ability of TiO_2_ over the wide spectrum of solar energy is still feeble and unsatisfactory.

Recently, elemental semiconductors, such as α-sulphur^6^ and red phosphorus (RP), have attracted considerable attention in the field of photocatalysis because of their unique advantages, such as wide visible light absorption ability, low cost, and earth abundance, which make it suitable for a range of practical applications^6^,^7^. Among them, RP has been reported to be a promising candidate for photocatalysis under the solar spectrum owing to its narrow band gap. Despite this, the photocatalytic efficiency of RP is limited due to the rapid recombination rate of photogenerated electrons and holes. Therefore, noble metal and carbon-based materials have been used as supporting and/or co-catalyst to solve this problem and improve its photocatalytic performance^8^,^9^,^10^. On the other hand, the development of RP-based nanohybrids for the environmental remediation is still limited because of the use of various chemicals and multiple synthetic processes, which limited their applications in the photocatalysis field^8^,^9^,^10^,^11^,^12^. Therefore, it is worthy to combine the fascinating properties of the RP, i.e., wide visible light absorption and low band gap, and TiO_2_, i.e., charge accumulating properties, which helps prolong the recombination lifetime, can be an effective way of fabricating highly visible light active nanohybrid materials.

In this study, the RP-TiO_2_ nanohybrids were prepared successfully via a one-step ball milling process using commercial RP and TiO_2_ nanoparticles as the precursors. The different weight percentages of RP and different milling times were investigated and optimized. The model organic pollutant, Rhodamine B (RhB), was decolorized by the RP-TiO_2_ nanohybrid under wide visible photoirradiation, whereas negligible performance was observed by the reference photocatalysts. In addition, the photoelectrochemical performance, such as electrochemical impedance spectroscopy (EIS), differential pulse voltammetry (DPV), and linear scan voltammetry (LSV) in the dark and under visible light irradiation were also investigated and studied in detail. The improved photodegradation and photoelectrochemical behavior of RP-TiO_2_ was attributed to the improved light absorption ability and effective separation of electrons and hole in RP-TiO_2_.

The RP-TiO_2_ nanohybrid photocatalysts was synthesized via the simple milling of commercially available RP powder and TiO_2_ with 50 wt. % RP (RP-TiO_2_) in a horizontal oscillatory mill. The RP-TiO_2_ was further milled for different times, *i.e.*, for 0 (RP-TiO_2_-0 h), 12 (RP-TiO_2_-12 h) and 24 (RP-TiO_2_-24 h) at 250 rotations per minute. The prepared RP-TiO_2_ was used for the characterization and photocatalytic activity studies. For a comparison study, TiO_2_ with 10 (RP-TiO_2_-1) and 20 wt. % (RP-TiO_2_-2) of RP were also prepared using the above mentioned method. A control photocatalyst was also prepared by hand grinding the same composition of TiO_2_ and RP (RP-TiO_2_-mix), as mentioned above.

## Result and Discussion

**Figure 1:**
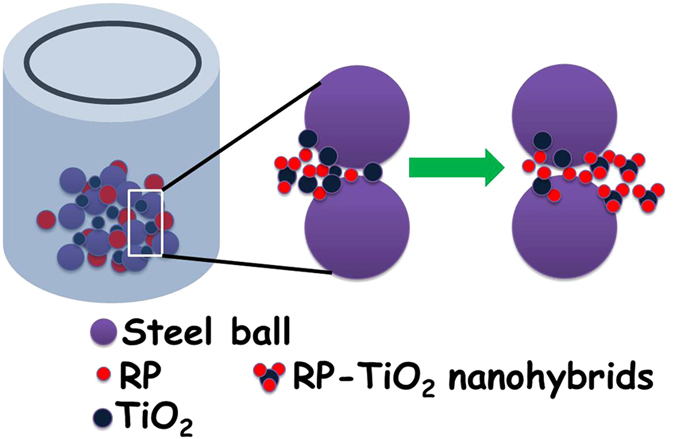
Proposed schematic diagram of the high energy ball milling synthesis mechanism for the fabrication of RP-TiO_2_ nanohybrids.

Figure 1 presents the proposed mechanism of phosphorus doping and its interactions in TiO_2_ during high energy ball milling. Generally, ball milling is used for the production of large scale nanosized materials because it effectively grinds large particles to the nano regime through the high energy forces developed during mechanical grinding. During the synthesis of the RP-TiO_2_ nanohybrid, strong mechanical energy was created by colliding high speed stainless steel balls with the reactant media and the cylindrical shell. As a result, the relevant high energy transfer through the hard phase materials with the reactants followed by a repetitive fracturing and welding process confirms that the milling process leads to a solid-state reaction of RP and TiO_2_, resulting in the formation of a nanohybrid photocatalyst^13^.

### XRD analysis

**Figure 2:**
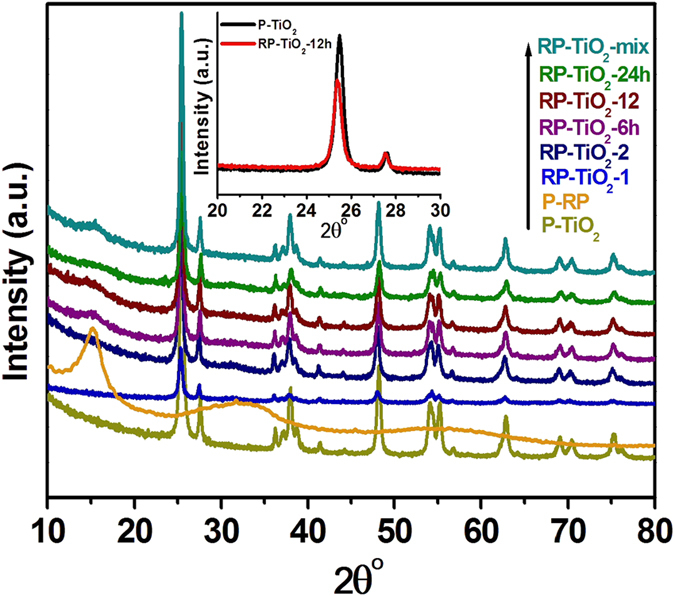
XRD patterns of the pure TiO_2_ (P-TiO_2_), commercially available pure red phosphorus (P-RP), TiO_2_ with 10 wt. % of RP (RP-TiO_2_-1), TiO_2_ with 20 wt. % of RP (RP-TiO_2_-2), TiO_2_ with 50 wt. % RP milled for 6 h (RP-TiO_2_-6 h), TiO_2_ with 50 wt. % RP milled for 12 h (RP-TiO_2_-12 h), TiO_2_ with 50 wt. % RP milled for 24 h (RP-TiO_2_-24 h), and TiO_2_ with 50 wt. % RP prepared by hand grinding (RP-TiO_2_-mix). The inset shows the shifting and broadening of the peak.

The crystal structure of the as-prepared RP-TiO_2_ nanohybrids was examined by XRD. The XRD pattern of the commercial red phosphorus (RP) showed a diffraction peak at 15° 2θ, which is the standard peak position of a medium-range ordered structure of phosphorus, whereas the XRD pattern of pure TiO_2_ (P-TiO_2_; Figure 2) displays the main characteristic diffraction peaks at 25.3°, 37.8°, 48.0°, 53.9°, 62.7°, and 75.0° 2θ, which were assigned to the (101), (004), (200), (105), (204), and (215) crystal planes of TiO_2_^2^,^3^,^4^,^14^. As shown in Figure 2, the XRD patterns of the TiO_2_ with 50 wt. % RP milled for 12 h (RP-TiO_2_-12 h) did not showing any new phases in the XRD pattern, which suggests that the composite consists of commercial RP and TiO_2_. Interestingly, a thorough examination of the XRD pattern revealed a shift in the peak positions of the RP-TiO_2_-12 h toward a lower angle compared to pure TiO_2_ (P-TiO_2_), which may be due to the existence of interactions between the TiO_2_ and phosphorus. In addition, the doping of phosphorus in the TiO_2_ lattice generates residual stress in the matrix, which leads to a shift of the characteristic diffraction angle of TiO_2_^15^,^16^.

### TEM and HR-TEM Analysis

**Figure 3:**
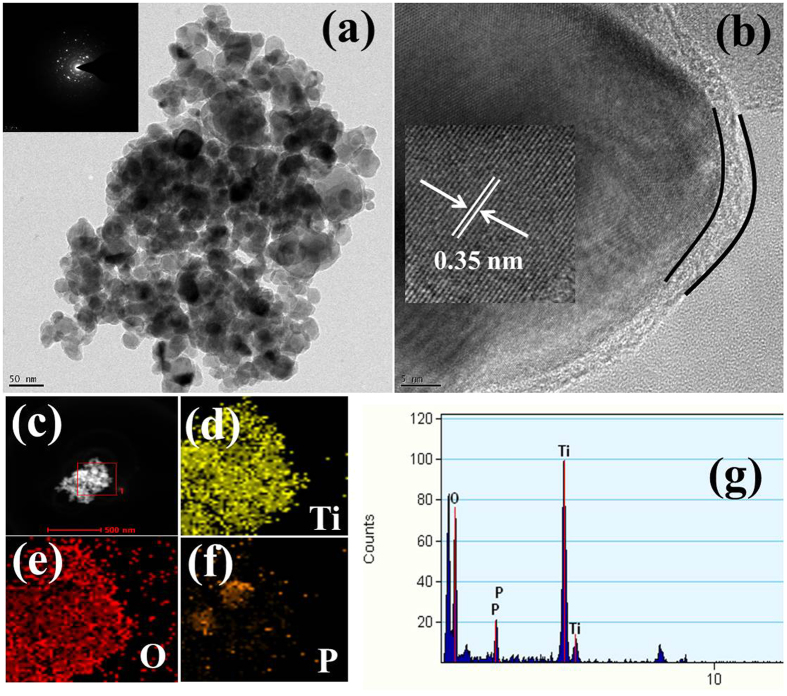
(**a**) TEM image and inset shows the SAED pattern, (**b**) HR-TEM image and inset shows the lattice fringe, (**c–f**) scanning transmission electron microscopy elemental mapping, and (**g**) EDX of the RP-TiO_2_-12 h nanohybrid.

To gain more insight into the interfacial interaction and improved charge separation efficiency over the RP-TiO_2_-12 h, TEM and HR-TEM was conducted and the results are shown in Figure 3. Figures S1 and S2 show HRTEM images and the SAED pattern of the P-TiO_2_. Figure 3a,b shows that the mean diameter of the RP-TiO_2_-12 h are ~25–30 nm. The acquired SAED pattern shows the well-crystalline behavior of the RP-TiO_2_-12 h. As shown in Figure 3, the corresponding HR-TEM image showed a high degree of structural uniformity and a well-ordered lattice structure of the RP-TiO_2_-12 h with an interplanar spacing of ~0.35 nm, which is well matched to that of the (101) crystal plane of TiO_2_. Moreover, there was a thin and intact coating of phosphorus observed on the TiO_2_ surface clearly showing the interface between TiO_2_ and phosphorus. In addition to the imaging mode of TEM, scanning transmission electron microscopy elemental mapping was also conducted to examine the elements present in the RP-TiO_2_-12 h, as shown in Figure 3c–f. The mapping results showed that Ti, O and P are present in RP-TiO_2_. Figure 3g shows the EDX spectra of the RP-TiO_2_-12 h, corresponding to the Ti (K), O (K) and P (K) lines, which are also in accordance with the mapping results.

### Photo-Physical Properties and Mobility Gap Calculation of the RP-TiO_2_ nanohybrid

**Figure 4:**
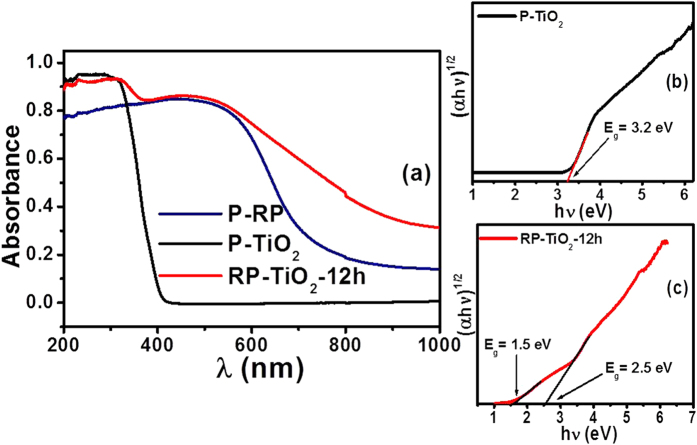
(**a**) Comparative UV-visible diffuse absorbance spectra of the P-RP, P-TiO_2_ and RP-TiO_2_-12 h, (**b**,**c**) plots of (αhν)^1/2^ *vs*. the energy of absorbed light of P-TiO_2_ and RP-TiO_2_-12 h.

The photophysical properties of the as-prepared RP-TiO_2_ nanohybrid photocatalysts were examined by UV-visible diffuse absorption and PL spectroscopy. Figure S3 shows the comparative UV-visible diffuse absorption spectra of pure TiO_2_ (P-TiO_2_), TiO_2_ with 10 wt. % of RP (RP-TiO_2_-1), TiO_2_ with 20 wt. % of RP (RP-TiO_2_-2), TiO_2_ with 50 wt. % RP milled for 6 h (RP-TiO_2_-6 h), TiO_2_ with 50 wt. % RP milled for 24 h (RP-TiO_2_-24 h), and TiO_2_ with 50 wt. % RP prepared by hand grinding (RP-TiO_2_-mix). Compared to P-TiO_2_, the other sample prepared with RP showed weak visible light absorption and its absorption band did not change as much as the optimized RP-TiO_2_-12 h photocatalyst (Figure 4a). In addition, the absorption spectra of the RP-TiO_2_-mix were also analyzed to determine the effect of ball milling on the absorption characteristics of TiO_2_. The absorption spectra displayed a similar absorption edge to the bare TiO_2_, which showed that there are no interactions between the RP and TiO_2_ and that mechanical grinding under optimized conditions can play an important role in improving the visible light absorption ability of TiO_2_. Figure 4a display the diffuse absorption spectra of the RP-TiO_2_-12 h nanohybrid, TiO_2_ nanoparticles, and P-RP. Compared to the other samples, the optical absorption of the RP-TiO_2_-12 h was enhanced substantially in the visible regime due to the light absorption characteristics of RP. The spectra also show that the absorption edge RP-TiO_2_-12 h is red shifted compared to P-TiO_2_. The red shifted absorption spectra provide some evidence of the interactions between RP and TiO_2_, which was confirmed further by the other characterization techniques and its photocatalytic behavior. The band gap of the RP-TiO_2_-12 h and P-TiO_2_ were obtained from the Kubelka-Munk functions versus the band gap energy plot and found to be ~3.2 eV for P-TiO_2_ and ~2.5 eV for the RP-TiO_2_-12 h nanohybrid (Figure 4b,c)^17^. On the other hand, an additional lower band gap of 1.5 eV was also observed in the case of RP-TiO_2_-12 h, which was attributed to the conduction band tail states arising from the impurity or doping that extends below the conduction band minimum. The significant band gap narrowing of the RP-TiO_2_-12 h was attributed to phosphorus doping into the TiO_2_ lattice, which leads to a decrease in the band gap of TiO_2_. Yang *et al.*^18^ and Gopal *et al.*^19^ reported that the red shift in the band gap of TiO_2_ is due to the substitution of pentavalent phosphorus in the Ti^4+^ sites^20^. These results suggest that the as-prepared RP-TiO_2_-12 h photocatalysts can be a highly visible light responsive photocatalysts compared to the bare materials, highlighting its potential applications in visible light-driven photocatalysis reactions.

**Figure 5:**
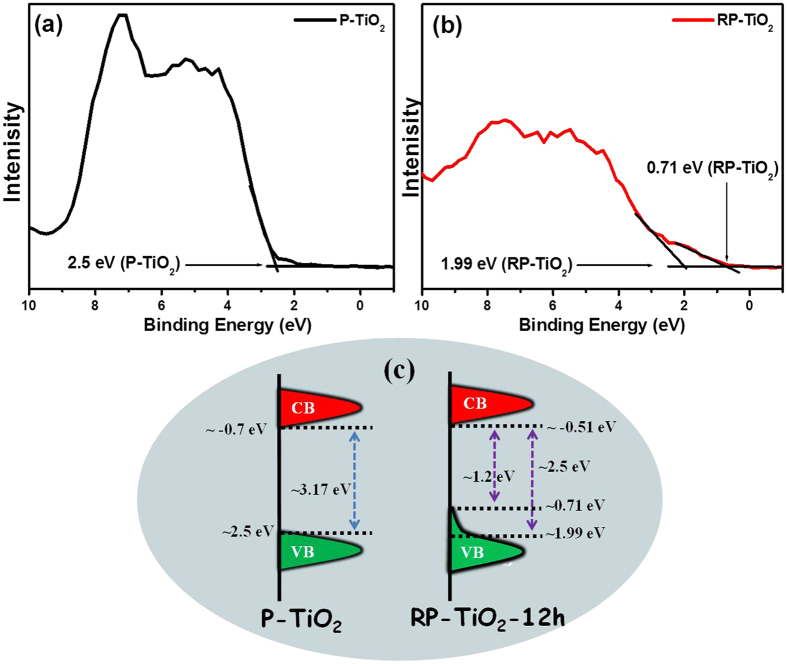
XPS valence band spectra of (**a**) P-TiO_2_, (**b**) RP-TiO_2_-12 h, and (**c**) schematic diagram of the DOS of RP-TiO_2_-12 h and P-TiO_2_.

To gain greater insight into the band gap reduction and improved photophysical properties of the RP-TiO_2_-12 h nanohybrid, the density of states (DOS) was determined from the UV-visible diffuse absorption results (Figure 4a–c) and valance band (VB) XP spectra (Figure 5). To obtain more insight and better understanding of the band gap narrowing phenomenon in RP-TiO_2_-12 h, valence band XPS was performed and density of electronic states of RP-TiO_2_-12 h and P-TiO_2_ was also obtained based on the VB XPS results and visible diffuse absorption spectroscopy results. P-TiO_2_ showed valence band DOS characteristics with the edge of the maximum energy at approximately 2.5 eV (Figure 5a), which is similar to previous reports, whereas the valence band maximum energy of RP-TiO_2_-12 h was estimated to be 1.99 eV followed by a band tail at ~0.71 eV (Figure 5b)^21^,^22^. The optical band gap energy of the P-TiO_2_ and RP-TiO_2_-12 h are 3.2 eV and 2.5 eV, respectively. Therefore, the conduction band (CB) minimum of the P-TiO_2_ and RP-TiO_2_-12 h would occur at ~ −0.67 eV and ~ −0.51 eV, respectively. Based on these results, the absorption onset in RP-TiO_2_-12 h was located at ~1.99 eV with a maximum energy associated with the band tail at ~0.71 eV. Therefore, substantial band gap narrowing of the RP-TiO_2_-12 h was observed, which was attributed to slight VB tailing^21^,^22^. A combination of these results with the optical band gap measurements suggests a decrease in the band of the TiO_2_. These results also suggest that the presence of RP in the RP-TiO_2_-12 h nanohybrids simultaneously shifts the valence band maxima and conduction band minima, which help reduce the band gap of TiO_2_. The decrease in band gap in this study may be due to the displacement and/or substitution of phosphorus in the TiO_2_ lattice system.

Figure 5c presents a schematic diagram of the DOS, which is proposed based on the combined results of the UV-visible diffuse absorption and VB XPS results. The valence band tail may extend above the valence band maxima or the up lifting of the valence band maxima, which might be responsible for the optical absorption onset in RP-TiO_2_-12 h^22^,^22^.

Photoluminescence spectroscopy was also proven to be a good tool for examining the photophysical properties of the material to better understand the fate of the electron-hole pairs, efficiency of charge carrier trapping, and recombination of electron holes pairs on the surface of the photocatalyst. In general, the strong emission intensity in the PL spectrum reflects the rapid charge recombination rate, whereas lower PL intensity means a lower electron-hole recombination rate, which is favorable for enhancing the photocatalytic activity of the materials^3^,^4^. Figure S4 shows that the PL spectra of the RP-TiO_2_-12 h and P-TiO_2_ provide similar excitonic PL signals with a similar curve shape, which shows that the presence of RP on TiO_2_ does not give any new PL emission. On the other hand, compared to P-TiO_2_, the PL emission intensity of RP-TiO_2_-12 h was totally suppressed due to the lower recombination rate of the photogenerated electrons and holes. This suggests that the presence of RP on the TiO_2_ surface might be responsible for improving the separation of photo-induced electrons and holes and suppressing recombination in the RP-TiO_2_-12 h nanohybrid. From the above discussion, it could be expected that RP-TiO_2_-12 possesses high photocatalytic activity compared to the bare materials.

**Figure 6:**
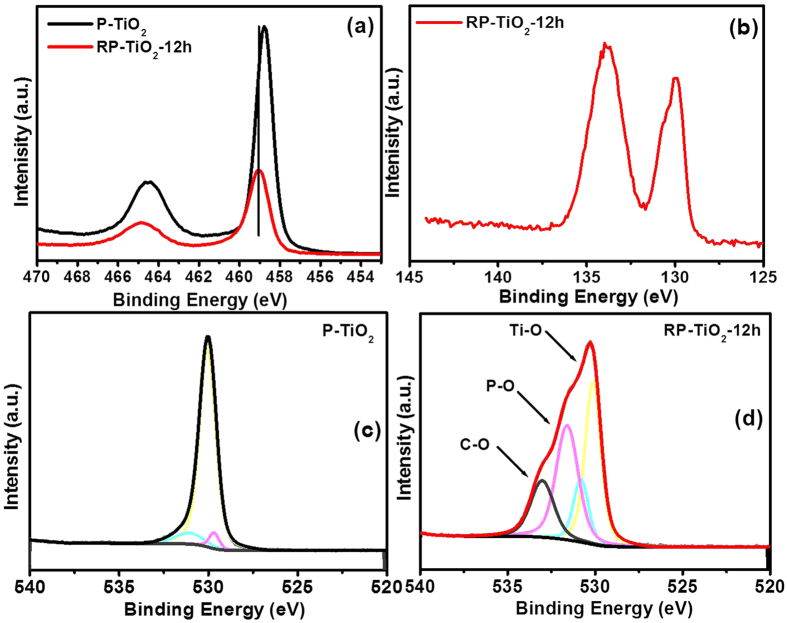
(**a**) Ti 2p photoelectron peak of P-TiO_2_ and RP-TiO_2_-12 h, (**b**) P 2p photoelectron peak of RP-TiO_2_-12 h, (**c**) O1 s photoelectron peak of P-TiO_2_, and (**d**) O 1 s photoelectron peak of RP-TiO_2_-12.

The chemical nature and elemental binding in RP-TiO_2_-12 h was investigated by XPS and compared with that of P-TiO_2_. The general scan spectra revealed the presence of Ti 2p, O 1 s, P 2p in the RP-TiO_2_-12 h nanohybrid (Figure S5). The high resolution spectra of the Ti 2p electron showed the two main peaks located at a binding energy (BE) of 458.76 ± 0.01 and 464.01 ± 0.01 eV for P-TiO_2_ , and 459.01 ± 0.01 and 464.8 ± 0.01 eV for RP-TiO_2_-12 h, which were assigned to Ti 2p_3/2_ and Ti 2p_1/2_ of TiO_2_, respectively (Figure 6a). The BE of Ti 2p in the RP-TiO_2_-12 h nanohybrid shifted to a higher value than P-TiO_2_, which provides strong evidence for the presence of an interfacial interaction between phosphorus and TiO_2_. The core-level spectrum of P 2p shows two peaks at 129.97 ± 0.01 eV and 130.62 ± 0.01 eV, which was attributed to the spin-orbit doublets of P 2p_3/2_ and P 2p_1/2_, whereas the peak at 134.4 ± 0.01 eV indicated phosphorus in the pentavalent oxidation sate (Figure 6b). According to previous reports, the ionic radii of P (0.38) is suitable for entering the Ti^4+^ (0.67) sites and formed Ti-O-P bonds. The formation of Ti-O-P bonds in the crystal lattice may explain the significant band reduction of TiO_2_ as well as the shift in the binding energy^19^. These results are also in accordance with the UV-visible diffuse absorption and XPS valance band results. The existence Ti-O-P bonds were identified further by the O 1 s high resolution XPS. The O 1 s photoelectron peak of P-TiO_2_ at a BE of ~530.05 ± 0.01 eV was assigned to the lattice oxygen in TiO_2_ (Figure 6c). As shown in Figure 6d, the deconvoluted O 1 s spectra peaks showed three different types of oxygen bonding in the RP-TiO_2_-12 h, including the Ti-O bonds, Ti-P-O bonds and C-O bonds, which further support the existence of chemical interactions between the TiO_2_ nanoparticles and phosphorus^23^.

### Confirmation and Proposed Mechanism for the Visible Light Photocatalytic Activity of RP-TiO_2_-12 h nanohybrid

**Figure 7:**
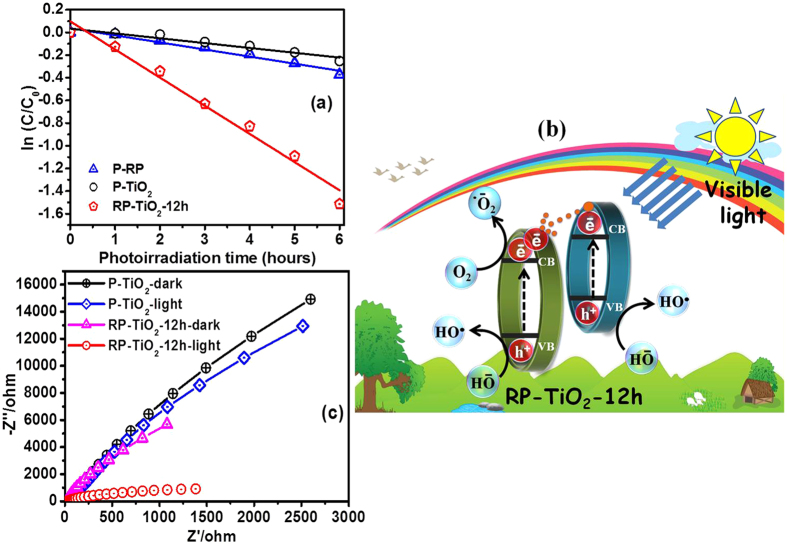
(**a**) Photodegradation kinetic plot of RhB as a function of the illumination time over P-TiO_2_ and RP-TiO_2_-12 h nanohybrid photocatalyst, (**b**) proposed schematic diagram of the photoexcited electrons-holes generation, separation, and their transport process over the RP-TiO2-12 h photocatalyst interface under visible light illumination, and (**c**) Nyquist plots.

The effects of the band gap reduction and interaction between RP and TiO_2_ during the mechanical milling process on the photocatalytic degradation of the model organic pollutant RhB, which is stable and used frequently in the photochemical and textile industries, were examined under dark and visible illumination conditions. A blank reaction with the catalyst in the dark (Figure S6) and without the catalyst in the light were conducted to determine the effects of the light and dark conditions on the photocatalytic degradation. The catalyst did not show activity under dark conditions. Moreover, self-degradation of RhB under illumination conditions was almost negligible. Figure 7a presents a photodegradation kinetic plot of RhB as a function of the illumination time, which was fitted to pseudo-first-order kinetics according to the literature^24^. As shown in Figure 7a, P-TiO_2_ showed negligible activity due to the wide band gap and high recombination rate of the photogenerated electrons and holes. This suggests that the P-TiO_2_ is not a good catalyst under visible illumination. In contrast to the other photocatalyst samples (P-RP, P-TiO_2_, RP-TiO_2_-1, RP-TiO_2_-2, RP-TiO_2_-mix, RP-TiO_2_-6 h, and RP-TiO_2_-24 h), which are shown in Figure S7, RP-TiO_2_-12 h showed significantly enhanced photocatalytic activity under visible light illumination. The other photocatalyst milled for longer time exhibited lower photocatalytic activity which was attributed to the agglomeration of fine particles due to the increased milling time, which leads to a decreased photocatalytic activity. The rate constant (*k*) was calculated to the level of increased activity of RP-TiO_2_-12 h compared to the bare material (Figure S7). The *k* values of P-RP, P-TiO_2_, RP-TiO_2_-1, RP-TiO_2_-2, RP-TiO_2_-mix, RP-TiO_2_-6 h, RP-TiO_2_-12 h, and RP-TiO_2_-24 h for the photocatalytic degradation of RhB under visible light irradiation were 0.06258/h, 0.04263/h, 0.02189/h, 0.01648/h, 0.02914/h, 0.04923/h, 0.2484/h, and 0.0716/h, respectively. The apparent rate constant of RP-TiO_2_-12 h for the degradation of RhB under visible light irradiation were almost four and six times higher than that of P-RP and P-TiO_2_. This improved photocatalytic performance of RP-TiO_2_-12 h was attributed mainly to the highly visible light harvesting ability due to its narrow band gap energy compared to P-TiO_2_, which could assist in the easy generation of electrons and holes over the RP-TiO_2_-12 h under visible light irradiation. In addition, an effective heterojunction formation at the RP and TiO_2_ interface may also play an important role in enhancing the effective separation of photogenerated electrons and holes, thereby improvement in visible light photocatalytic activity. The improvement in the visible light absorption region alone cannot ensure high photocatalytic activity because the separation of the photogenerated electron-hole pairs and their migration to the surface reaction sites also plays an important role in determining the photocatalytic performance. The recycling ability of the RP-TiO_2_-12 h after the catalytic reaction was observed by collecting, washing and drying the catalysts. The resulting photoctalyst showed the good stability after the three successive cyclic runs which further indicates its reusability (Figure S8).

Based on the enhanced performance shown by RP-TiO_2_-12 h, the possible photo excitation of electrons and holes and its electronic transfer over the surface of photocatalysts under visible illumination was proposed, as depicted in Figure 7b. The photocatalytic performance shown by P-TiO_2_ was considered to be negligible under visible light illumination due to the wide band gap and high recombination rate of the photo-induced electrons and holes. This phenomenon was also confirmed by the photocatalytic test, as discussed above. Compared to the other samples, RP-TiO_2_-12 h exhibited remarkably enhanced photocatalytic activity under similar conditions. This significant difference in the activity of the RP-TiO_2_-12 h could be explained in two parts because the high photocatalytic activity is not only due to the improvement in the light absorption ability of the photoctalysts available reaction sites also plays an important role in determining the photocatalytic performance. First, when RP-TiO_2_-12 h was irradiated with visible light, the photo excited electrons from the VB of TiO_2_ migrate to the CB of TiO_2_ easily due to its sufficiently narrow band gap. In addition, the VB electrons of RP also migrate to the CB of RP under similar conditions. These excess photoexcited electrons that accumulated on the surface of the TiO_2_ were then trapped by the dissolved oxygen molecules in water to yield superoxide radical anions (^•^O_2_^−^) and hydroxyl radicals (HO^•^)^25^. Similarly, the photogenerated holes present on the surface of TiO_2_ and RP react with the surface adsorbed hydroxyl ions to form highly reactive HO^•^. These highly reactive radicals are responsible for the photodegradation and mineralization of pollutants.

The position of the CB edge of the RP is more negative than TiO_2_, which is helpful for transferring the photoexcited electrons readily from the RP to the CB of TiO_2_ through the interface. In addition, the coating of TiO_2_ by RP might also be responsible for the absorption of visible light and therefore produces electron under visible photoirradiation, which is then further transfer to the CB of the TiO_2_. This phenomenon also supported the presence of RP on the surface of TiO_2_. Therefore, the direct transfer of the charge carrier may reduce the probability of the recombination of photogenerated species, which facilitates efficient charge separation and enhances the photocatalytic activity, as shown in Figure 7b. RP-TiO_2_-12h exhibited significantly enhanced photocatalytic activity compared to the other samples under the same conditions, suggesting that RP-TiO_2_-12 h exhibits stronger ability in the separation of electron-hole pairs, as shown by PL analysis (Figure S4). In addition, the heterojunction also plays an important role in the separation of photogenerated electrons and holes. On the other hand, the interaction between the RP and TiO_2_ through Ti-O-P could also introduce shallow traps at the surface or interface of TiO_2_ and further reduce the band gap, which helps enhance the visible light absorption ability of the materials. These shallow trap states can enhance the efficiency of the charge carriers between the interface, which helps increase the life time of the photogenerated electrons and holes, thereby improving the photocatalytic activity of RP-TiO_2_-12 h under visible light irradiation. Therefore, the superior photocatalytic activity of RP-TiO_2_-12 h can be attributed to the combined synergistic effects, such as the large number of charge carriers accumulated on the surface of the photocatalyst used to the catalyzed series of reduction and oxidation reaction, interfacial transfer of charge carriers, efficient charge separation and reduced band gap.

EIS measurements were taken under dark and under illumination conditions to examine the interfacial characteristic of the prepared P-TiO_2_ and RP-TiO_2_-12 h-based photoelectrodes, such as charge transfer resistance and recombination rate of the photogenerated electrons-holes. As shown in Figure 7c, the EIS result of RP-TiO_2_-12 h reflects the smaller arc radius compared to the P-TiO_2_, which suggests the better separation of the photogenerated electron-hole pairs and faster interfacial charge transfer under visible light irradiation over the RP-TiO_2_-12 h nanohybrid photoelectrode. In general, the existence of a smaller arc radius in the EIS plot demonstrates smaller electron transfer resistance, effective separation of photogenerated electron-hole pairs and faster interfacial charge transfer at the surface of the photoelectrodes^25^. These results reveal the analogous trend with PL analysis and photocatalytic activity.

**Figure 8:**
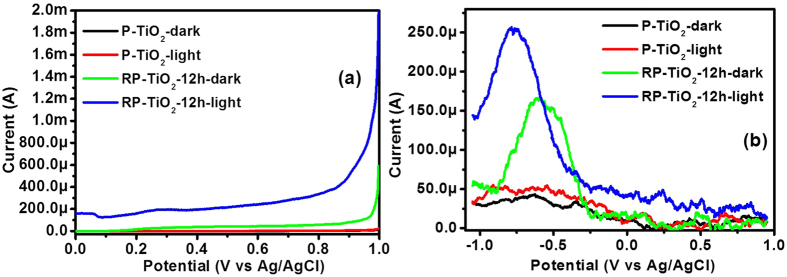
(**a**) Linear scan voltammograms and (**b**) DPV voltammograms obtained for P-TiO_2_ and RP-TiO_2_-12 h nanohybrid photoelectrodes in the dark and under visible light illumination.

LSV was conducted to obtain the photo responsive characteristics of the P-TiO_2_ and RP-TiO_2_-12 h electrodes in the dark and under illumination conditions^26^. Figure 8a shows that the P-TiO_2_ nanoparticle photoelectrode exhibited a poor photocurrent response, which was improved significantly after the addition of visible light responsive RP. The photocurrent of the RP-TiO_2_-12 h was approximately 2 fold higher than that of P-TiO_2_. The enhancement of the photocurrent of RP-TiO_2_-12 h might be due to the narrow band gap, wide visible light absorption ability and improved charge separation of photogenerated electron-hole pairs. In addition, the formation of a heterojunction at the metal-metal oxide interface may also play an important role in enhancing the effective separation of photogenerated electrons and holes, thereby increasing the photocurrent.

In addition to the charge carrier inducing photocatalytic reactions, these visible light photoinduced charge carriers accumulated on the surface of the photocatalysts are also responsible for inducing charging behavior in the composite materials^4^,^27^,^28^. A representative accumulative charge carrier and its charging behavior on the RP-TiO_2_-12 h nanohybrid was observed by DPV under dark and visible light illumination conditions and was compared with that of pure TiO_2_ (Figure 8b). Under visible light illumination, TiO_2_ exhibited almost no response due to the wide band gap. In contrast, the RP-TiO_2_-12 h nanohybrid showed an intense and well-defined quantized capacitance charging peak under visible light irradiation. The enhanced charge storage performance of the RP-TiO_2_-12 h nanohybrid over P-TiO_2_ can be attributed mainly to phosphorus, which induces band gap narrowing, and subsequently favors more visible light harvesting property. In other words, the synergistic effects of the light harvesting ability of RP and reduced band gap of TiO_2_ dramatically improve the photoinduced charge carrier generation over the surface of the photocatalyst under visible light illumination leading to its excellent charge storage properties. These results highlight the analogous trend with PL analysis and the photocatalytic activity of the RP-TiO_2_-12 h.

## Conclusions

A highly visible light responsive RP-TiO_2_-12 h nanohybrid was prepared through a simple, cost effective and productive mechanical ball milling process. The mobility band gap was determined from the VB and UV-visible spectroscopy, and O 1 s and Ti 2p XPS provided evidence of an interfacial interaction through chemical bonding between RP and TiO_2_. In addition, doping the TiO_2_ lattice with red phosphorus results in enhanced visible light absorption of the RP-TiO_2_-12 h. As a result, RP-TiO_2_-12 h achieved significantly enhanced photocatalytic degradation ability for the degradation of the model pollutant compared to the P-TiO_2_ and P-RP under visible light irradiation. Furthermore, studies in the dark and under visible light conditions conclusive evidence of the effective separation and life time of the photogenerated charge carriers as well as the enhancement of the visible light activity of RP-TiO_2_-12 h. The combined synergistic effects, including interfacial interaction and interface bond formation through the Ti-O-P and resulting narrow mobility band gap were found to be the main reasons for the significant enhancement in the photodegradation rate and the photoelectrochemical performance of the RP-TiO_2_-12 h.

## Experimental

### Materials

TiO_2_ nanoparticles and RhB were purchased from Sigma Aldrich, whereas RP was obtained from Yakuri Pure Chemicals, Kyoto Japan. Ethyl cellulose and α-terpineol (C_10_H_18_O) were supplied by KANTO Chemical Co., Japan. Sodium sulfate (Na_2_SO_4_) was acquired from Duksan Pure Chemicals Co. Ltd. South Korea. The fluorine-doped transparent conducting oxide glass substrates (FTO; F-doped SnO_2_ glass; 7 Ω/sq) were acquired from Pilkington, USA. The other chemicals used in this study were of analytical grade and the solutions were prepared in deionized water obtained from a PURE ROUP 30 water purification system.

## Methods

Phase characterization was analyzed by X-ray diffraction (XRD, PANalytical, X’pert PRO-MPD, Netherland) using Cu Kα radiation (λ = 0.15405 nm). The photophysical properties of the samples were measured by ultraviolet-visible-near infrared (UV-VIS-NIR, Cary 5000, VARIAN, USA) spectrophotometry equipped with a diffuse reflectance integrating sphere. The photoluminescence (PL, Kimon, 1 K, Japan) spectra of the samples were recorded with excitation wavelength of 325 nm and range 200-800 nm. PL was conducted at the Korea Basic Science Institute, Gwangju Center, South Korea. The chemical and binding state of the sample was examined by X-ray photoelectron spectroscopy (XPS, ESCALAB 250 XPS System, Thermo Fisher Scientific U.K.) using monochromatized Al Kα x-rays (hν = 1486.6 eV). The surface characteristics and crystalline structure of the nanohybrid were observed by field emission transmission electron microscopy (FE-TEM, Tecnai G2 F20, FEI, USA) at an accelerating voltage of 200 kV. The photoelectrochemical and photocatalytic experiments were performed using a 400 W lamp, which provided the visible light with an intensity of 31 mW/cm^2^ (λ > 500 nm, 3 M, USA). EIS was performed using a three electrode cell with a 0.2 M Na_2_SO_4_ electrolyte using a potentiostat (VersaSTAT 3, Princeton Research, USA). The working electrodes were prepared using a previously reported method^4^. Briefly, 100 mg of RP-TiO_2_ was dissolved in a mixture of ethyl cellulose and alpha terpinol, and the resulting solution was mixed well and coated onto a FTO glass electrode using the doctor blade method and RP- coated (FTO) glass was used as the working electrode, whereas Ag/AgCl (3 M KCl) along with the inserted Pt were used as the reference and counter electrodes, respectively. Mechanical milling was performed using a ball milling machine (XQM-0.4A, Changsha Tianchuang Powder Technology Co. Ltd, China).

### Photocatalytic Test

The photodegradation ability test of the RP-TiO_2_ photocatalyst was evaluated by the decolorization of a model organic pollutant, i.e., RhB under visible photoirradiation. The photodegradation experimental process was similar to a previous report^3^,^4^. Briefly, 5 mg of the photocatalyst was dispersed in 20 mL of an aqueous solution of RhB and stirred for 10 min to achieve adsorption-desorption equilibrium. After that, the above suspensions were irradiated with visible light to initiate the photocatalytic degradation reaction. At certain time, a 2 mL aliquot was taken and further centrifuged to separate the catalyst from the aliquot. The absorbance of the above centrifuged solution was recorded using an Optizen 2120UV UV–vis spectrophotometer. The absorption data was used to calculate the photocatalytic efficiency of the RP-TiO_2_ photocatalyst. The calculation methodology was similar to previous reports^3^,^4^.

### Photoelectrochemical Studies (EIS, LSV and DPV)

The photoelectrochemistry of the prepared RP-TiO_2_ and P-TiO_2_ electrodes were measured using EIS, DPV and LSV in the dark and under visible photoirradiation. The EIS and LSV experiments were performed in an aqueous electrolytic solution of Na_2_SO_4_ (0.2 M, 50 mL). LSV was recorded in the dark and under visible photoirradiation at a scan rate of 50 mV/s over a potential range of −0.9 to +0.9 V, whereas EIS was first performed in the dark and later under visible light irradiation at 0.0 V and frequencies ranging from 1 to 10^4^ Hz. DPV was performed with a pulse height of 50 mV, pulse width of 0.005 s and scan rate of 4 mV/s.

### Preparation of RP-TiO_2_ Nanohybrids Photocatalysts

The RP-TiO_2_ nanohybrid photocatalysts was synthesized via the simple milling of commercially available RP powder and TiO_2_ with 50 wt. % RP (RP-TiO_2_) in a horizontal oscillatory mill. The RP-TiO_2_ was further milled for different times, *i.e.,* for 0 (RP-TiO_2_-0 h), 12 (RP-TiO_2_-12 h) and 24 (RP-TiO_2_-24 h) at 250 rotations per minute. The prepared RP-TiO_2_ was used for the characterization and photocatalytic activity studies. For a comparison study, TiO_2_ with 10 (RP-TiO_2_-1) and 20 wt. % (RP-TiO_2_-2) of RP were also prepared using the above mentioned method. A control photocatalyst was also prepared by hand grinding the same composition of TiO_2_ and RP (RP-TiO_2_-mix), as mentioned above.

## Additional Information

**How to cite this article**: Ansari, S. A. and Cho, M. H. Highly Visible Light Responsive, Narrow Band gap TiO_2_ Nanoparticles Modified by Elemental Red Phosphorus for Photocatalysis and Photoelectrochemical Applications. *Sci. Rep.* **6**, 25405; doi: 10.1038/srep25405 (2016).

## Supplementary Material

Supplementary Information
