# Supplementary material for: Highly Visible Light Responsive, Narrow Band gap TiO2 Nanoparticles Modified by Elemental Red Phosphorus for Photocatalysis and Photoelectrochemical Applications
Source: Sci Rep. 2016 May 5;6:25405. doi: 10.1038/srep25405 (PMC4857111; doi:10.1038/srep25405)
Supplement: Supplementary Information [file srep25405-s1.pdf]

Supporting Information

Highly Visible Light Responsive, Narrow Band gap TiO_2 Nanoparticles Modified by Elemental Red Phosphorus for Photocatalysis and Photoelectrochemical Applications

Sajid Ali Ansari* **and Moo Hwan Cho***

School of Chemical Engineering, Yeungnam University, Gyeongsan-si, Gyeongbuk 712-749, South Korea, Phone: +82-53-810-2517; Fax: +82-53- 810-4631.

*Corresponding authors: sajidansari@ynu.ac.kr, mhcho@ynu.ac.kr

HR-TEM Image of the P-TiO_2

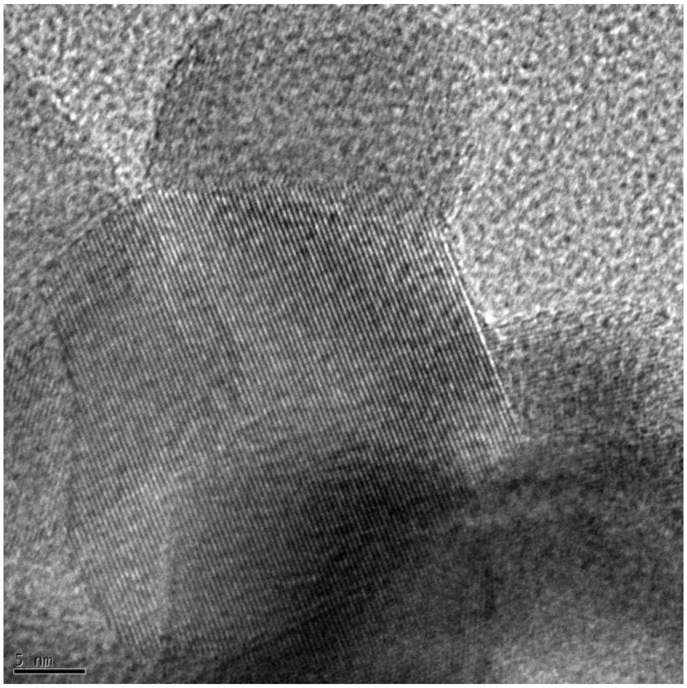

Figure S1. HR-TEM image of the P-TiO_2.

SAED Pattern of the P-TiO_2

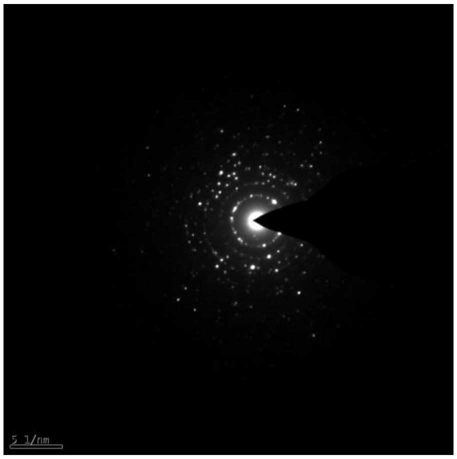

Figure S2. SAED pattern of the P-TiO_2.

UV-vis Diffuse absorption spectra of the P-TiO_2, RP-TiO_2-1, RP-TiO_2-2, RP-TiO_2-6h, RP-TiO_2-24h, and RP-TiO_2-mix

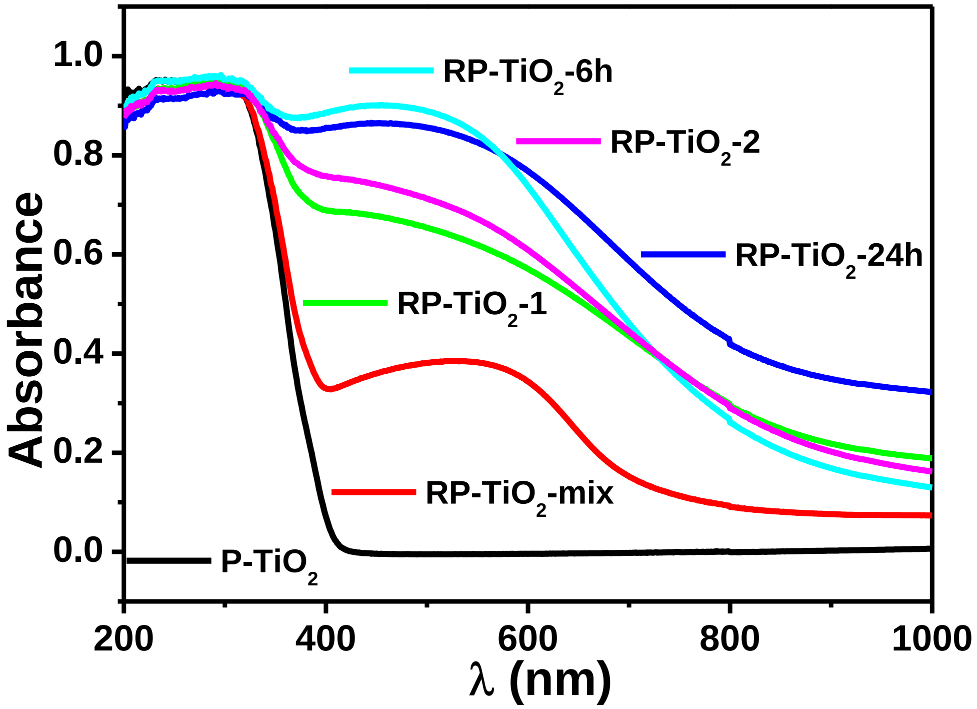

Figure S3. UV-vis Diffuse absorption spectra of the P-TiO_2, RP-TiO_2-1, RP-TiO_2-2, RP-TiO_2-6h, RP-TiO_2-24h, and RP-TiO_2-mix.

PL spectra of P-TiO_2 and RP-TiO_2-12h

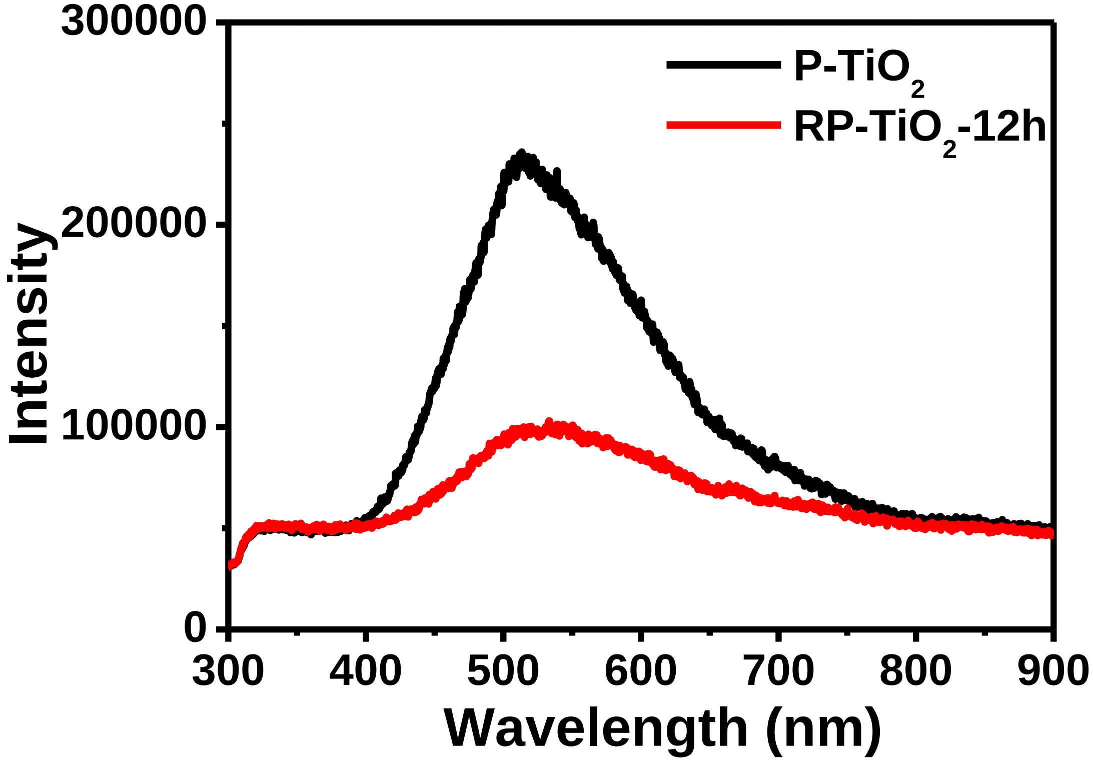

Figure S4. PL spectra of P-TiO_2 and RP-TiO_2-12h.

XPS survey spectra

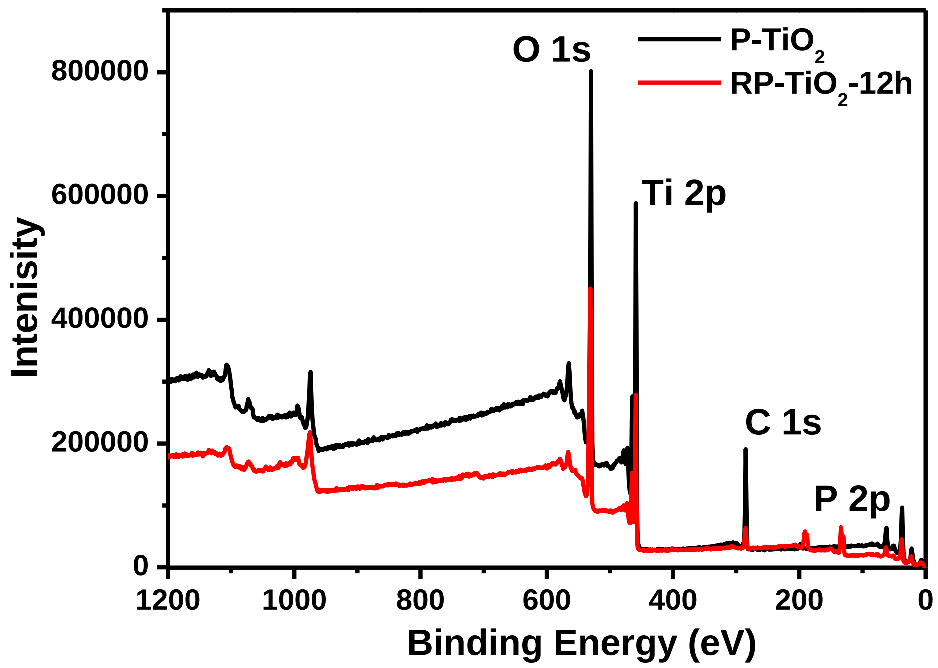

Figure S5. XPS survey spectra of the P-TiO_2 and RP-TiO_2-12h nanohybrid.

Dark reaction (with catalyst) and light reaction (without catalyst)

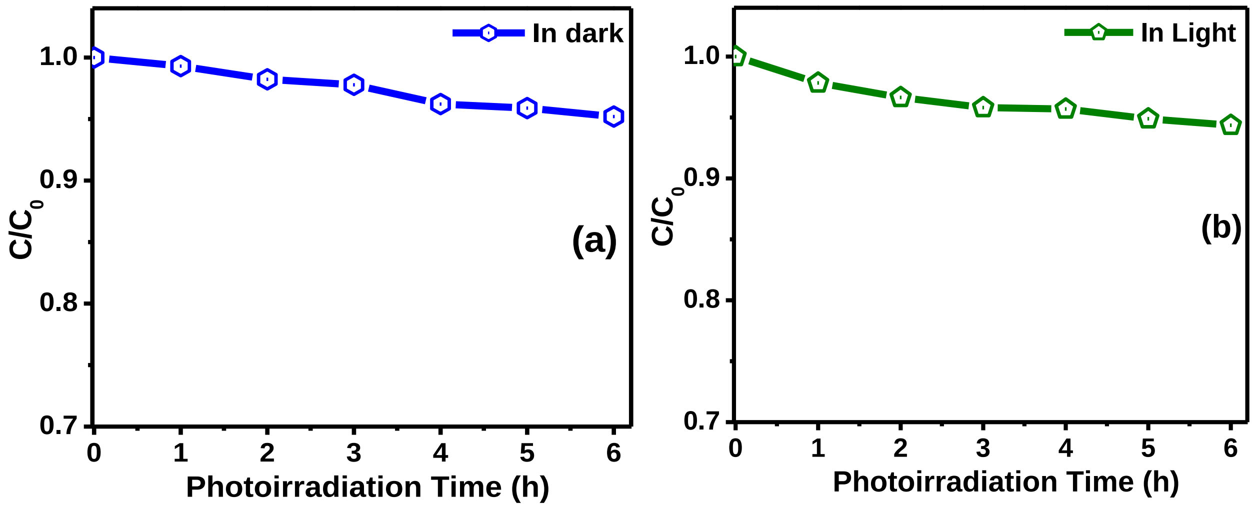

Figure S6. The C/C_0 versus irradiation time (h) plots for RhB degradation (**a**) Dark reaction (with catalyst) and (**b**) light reaction (without catalyst).

Photodegradation kinetic plot of RhB as the function illumination time

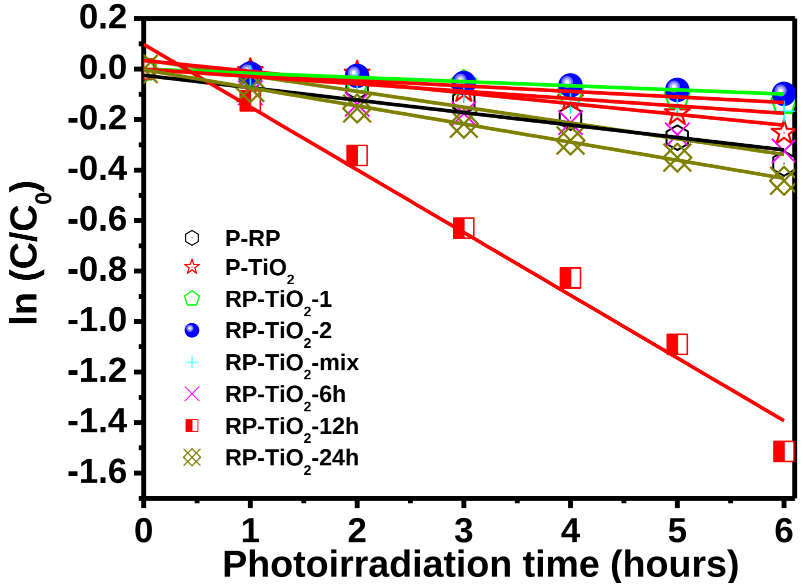

Figure S7. Plot of ln C/C_0 vs the photoirradiation time for the photodegradation of RhB by P-TiO_2, P-RP, RP-TiO_2-1, RP-TiO_2-2, RP-TiO_2-6h, RP-TiO_2-12h, RP-TiO_2-24h, and RP-TiO_2-mix.

Cyclic Stability

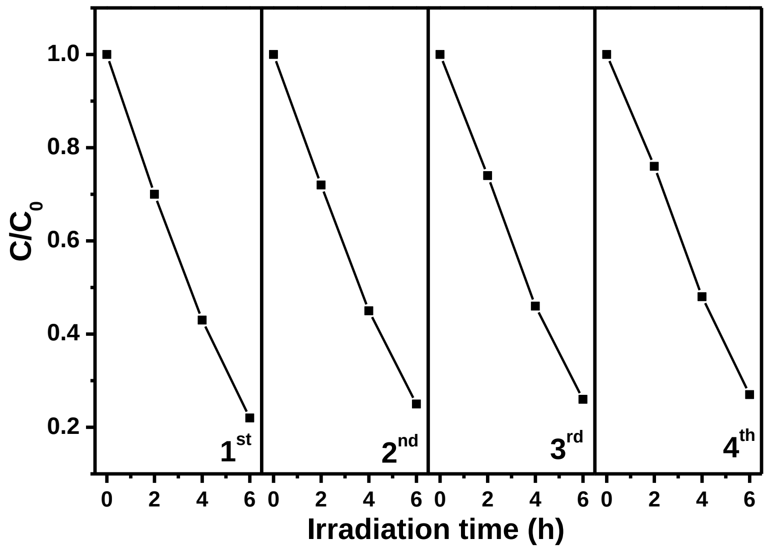

Figure S8. Stability test results of RP-TiO_2-12h.
